# The Art of Intraoperative Glioma Identification

**DOI:** 10.3389/fonc.2015.00175

**Published:** 2015-07-30

**Authors:** Zoe Z. Zhang, Lisa B. E. Shields, David A. Sun, Yi Ping Zhang, Matthew A. Hunt, Christopher B. Shields

**Affiliations:** ^1^Department of Neurosurgery, University of Minnesota, Minneapolis, MN, USA; ^2^Norton Neuroscience Institute, Norton Healthcare, Louisville, KY, USA; ^3^Department of Anatomical Sciences and Neurobiology, University of Louisville School of Medicine, Louisville, KY, USA

**Keywords:** glioma, tumor, resection, technique, intraoperative

## Abstract

A major dilemma in brain-tumor surgery is the identification of tumor boundaries to maximize tumor excision and minimize postoperative neurological damage. Gliomas, especially low-grade tumors, and normal brain have a similar color and texture, which poses a challenge to the neurosurgeon. Advances in glioma resection techniques combine the experience of the neurosurgeon and various advanced technologies. Intraoperative methods to delineate gliomas from normal tissue consist of (1) image-based navigation, (2) intraoperative sampling, (3) electrophysiological monitoring, and (4) enhanced visual tumor demarcation. The advantages and disadvantages of each technique are discussed. A combination of these methods is becoming widely accepted in routine glioma surgery. Gross total resection in conjunction with radiation, chemotherapy, or immune/gene therapy may increase the rates of cure in this devastating disease.

## Introduction

The purpose of brain-tumor resection is to maximize tumor removal while sparing healthy tissue. The extent of resection is a key prognostic factor; however, complete tumor resection is often not possible. Improvements in survival time, functional recovery, and tumor recurrence rates are associated with increasing extents of safe resection in older patients (≥60 years) (1). Due to the imprecise correlation between pre-operative images and intraoperative anatomy as well as poor differentiation of low-grade glioma from normal tissue in non-eloquent areas, substantial tumor volume may remain postoperatively. The frequency of residual tumor following surgery is surprisingly high, leading to rapid disease recurrence (2, 3).

To avoid these shortcomings, better delineation of normal from tumor tissue intraoperatively could improve the clinical outcomes following tumor removal surgeries. Delineation tools and methods have been designed and improved continuously to increase the chance of total tumor resection and to decrease normal tissue damage adjacency of important structures. Intraoperatively, certain gliomas may be vaguely suggested by their physical differences, such as natural color or the dissimilar firmness of the tumor tissues. The usefulness of these physical signs for recognition of glioma also depends on the location and extension. If the glioma is superficially located, the affected gyri may become distended, edematous, discolored, or possess different vascularity. These signs are less obvious in deep-seated lesions.

Traditionally, excision of gliomas relies on the neurosurgeon’s ability to detect slight variations in cortical topography; however, even the experienced surgeon may be unable to detect the changes. Because the limited differentiable capabilities of naked eyes and hands of the surgeons, advanced imaging methods and identification assays are in continual development for glioma delineation. Various technologies may detect subtle differences between the glioma and normal tissue using, such modalities as ultrasound (US), computed tomography (CT), nuclear magnetic resonance imaging, functionally by electrophysiological assays (4), and biologically by assessments on protein, lipid, DNA, ion channel, vascular permeability, autofluorescence, and metabolism alterations. In this review, the representative methods and tools for differentiating glioma from normal CNS tissue are discussed.

## Intraoperative Delineation of Gliomas

### Image-based navigation

Delineation of gliomas is initiated during pre-operative surgical planning. Gross tumor delineation is achieved using imaging technologies based on US (reflecting ultrasonic waves), computed tomographic x-ray, and magnetic resonance of tissue. Pre-operative and intraoperative imaging confers anatomic landmarks that help determine the surgical approach.

#### MRI Navigation

MRI navigation is an essential tool in surgical planning. Both CT and MRI are used for surgical navigation; however, MRI is preferred due to superior visualization of both the tumor and the normal anatomy in most cases. MRI neuronavigation assists the surgeon in determining the optimal approach and monitoring the extent of tumor resection intraoperatively. Several intraoperative systems are available, such as Brainlab (Feldkirchen, Germany), CranialMap (Stryker, Kalamazoo, MI, USA), StealthStation (Medtronic, Minneapolis, MN, USA), each differing in hardware and software design. Neuronavigation systems incorporate multiple registration techniques to bring the imaging information into the surgical field to assist surgical guidance. Regardless of the pros and cons of the various systems, frameless navigation may be superior to frame-based ones because image distortion may occur in an area of interest situated adjacent to the frame (5). High-field MRI coupled with stereotactic neuronavigation provides anatomical and functional guidance in glioma surgery (6–8). Diffusion tensor imaging defines white matter fiber tracts to assist in identifying areas of tumor infiltration and reducing damage to normal tracts during tumor excision (9). Neuronavigation with multimodal imaging data, such as structural and metabolic data, fiber tracking, and 3D visualization, has been proposed to optimize the safety and extent of tumor resection (10).

Based on information obtained from pre-operative images, the accuracy of navigation may be influenced by brain sagging due to head position, CSF drainage, and the effects of tissue resection. Neuronavigation using intraoperative MRI imaging may avoid potential errors caused by tissue shift (11, 12). Adaptation of MRI scans to the surgical suite increases the accuracy of tumor delineation and the extent of glioma resection. The incidence of complete tumor resection was significantly higher using intraoperative MRI with no increase in neurological deficits compared to conventional surgery (*p* = 0.023) (13, 14). The intraoperative MRI is inconvenient due to its bulky coil that encroaches on limited surgical space, and its strong magnetic field requires MRI compatible tools and supplies when surgery is performed in the magnetic field (15, 16). Although the ioMRI scanner in which the patient is fixed in one position has several advantages (no need to move the patient during the operation, which could lead to potential anesthetic complications, a shorter time to obtain images), it requires non-ferro-magnetic instruments that are not as robust as conventional neurosurgical instruments. Most available ioMRI scans require that the patient be moved from the surgical site located outside the magnetic field into the intraoperative scanner. Alternatively, the MR scanner may be moved to the patient who remains in the surgical site. These methods delay the surgery while the patient (or MRI scanner) is moved to the location in which images will be performed. However, having the surgical site remote from the scanner is advantageous insofar as conventional neurosurgical instruments may be used and a higher power MR scan is usually available. Intraoperative MRI may also be limited in tumor delineation during surgery in that surgery itself may induce contrast enhancement. This enhancement may make identification of residual tumor difficult or give the appearance of residual tumor rather than normal brain. A technique that improves repeat intraoperative imaging includes iron oxide nanoparticles (17).

#### Ultrasound Navigation

Ultrasound navigation is cost-effective and has been used intraoperatively for decades (18). US navigation is a real-time method for delineating solid, cystic, and necrotic tissues based on their different acoustic impedances and reflection coefficients. Three-dimensional ultrasonography has been widely adapted for placement of catheters, needles, or other instruments into sites of abnormal tissue (19). US is optimal for accurate placement of needles for glioma biopsy. Under its guidance, the trajectory and the depth of the biopsy needle may easily be planned. Intraoperative US imaging also helps to determine the surgical corridor and to guide tumor resection based on the size, shape, and localization of lesions *in situ* in real-time (20, 21). Newly developed linear array intraoperative US has a significantly superior ability to detect residual glioma compared to conventional intraoperative US. Linear array US rivals ioMR scans in identifying residual tumor; however, ioMRI has a higher specificity and lower sensitivity (22).

Contrast-enhanced US is used in neurosurgery to assist in the delineation of tumor margins. It also identifies afferent and efferent blood vessels as well as the perfusion patterns of the tumor, which is particularly valuable in highly vascular tumors (23, 24). Gross tumor resection of intracerebral high-grade tumors was achieved in 21/22 (95.5%) of patients with little morbidity using high-frequency US (hfioUS) (25). This method allowed detailed discrimination between normal, pathological, and edematous tissue in all patients.

The true border of the tumor is usually larger than that depicted by ultrasonography. In a study of 101 supratentorial glioma cases, intraoperative MRI proved to be more accurate in tumor delineation than intraoperative ultrasonography (26). The drawback of US is the inability to provide both high resolution and deep penetration. Improving resolution of ultrasonography may increase the precision of tumor delineation; however, signal accuracy deteriorates when identifying deep-seated tumors (27, 28).

### Intraoperative sampling

This method produces the greatest diagnostic accuracy although information from specimens from a single patient is spatially and temporally discontinuous. The final diagnosis, particularly for unusual tumors, may be delayed for several days and requires that specimens be placed together. Precision of tumor identification increases with the number of tissue samples obtained. Tumor differentiation is based on molecular, cellular, and structural differences between gliomas and normal tissue.

#### Mass Spectrometry-Based Analysis

Mass spectrometry molecular analysis is faster and more accurate compared to histological evaluation. The diagnosis is based on tumor-specific molecules and analysis of oncometabolites. Using desorption electrospray ionization mass spectrometry (DESI-MS), differences of lipids and their concentration between gliomas and normal brain are used to confirm the tumor type and to define tumor margins (29, 30). Due to its ability to rapidly analyze tissue samples, intraoperative mass spectrometry may confirm brain-tumor pathology (30–32).

### Electrophysiological monitoring (mapping)

Dielectric properties of human gliomas and surrounding tissues may be measured by a network analyzer using a coaxial line capacitive sensor. The permittivity and conductivity of tumors is 30% higher than surrounding normal tissue (33). These electrophysiological differences have not been used clinically to differentiate glioma types. Current electrophysiological methods used for intraoperative tumor delineation are indirect and define boundaries of normal circuitry adjacent to the glioma. Intraoperative functional mapping by electro-cortical stimulation or magnetoencephalography guides supratentorial glioma resection, especially valuable when the tumor is in proximity to eloquent brain. Functional mapping by applying lower electrical stimuli at either the cortical or subcortical levels identifies the eloquent areas before and during glioma resection, which improves the accuracy of tumor resection, reduces neurological deficits, and prolongs survival (34–39).

Although this method provides indirect evidence of tumor demarcation, its immediate feedback to the surgeon may avoid potential risks. A major shortcoming of this technique is that the patient must be awake to assess sensorimotor, language, calculation, and semantic function, especially for surgery on the dominant hemisphere (40).

### Visually enhanced methods for tumor demarcation

Tumor visualization may be enhanced using specially designed microscopes with filters capable of detecting fluorescent light emission with or without enhancement. The differentiation between tumor and normal tissue depends on the uptake of specific enhancing agents, metabolism, and the production of detectable bio-products by tumor. These differences are visualized under the surgical microscope equipped with appropriate filters. This is a promising strategy for real-time tumor discrimination *in situ*. Dye-based methods have been investigated but were unacceptable for clinical use due to inadequate color contrast between tumor and normal brain tissue (41). New microscope imaging technologies and novel enhancing agents continue to evolve, which provide a sharper delineation between tumor and normal brain tissue.

#### 5-Aminolevulinic Acid

5-Aminolevulinic acid (5-ALA) is an accepted method that enhances glioma visualization under blue light. The metabolism of 5-ALA is dependent on heme biosynthetic pathways. Since gliomas are deficient in ferrochelatase enzyme, accumulation of the fluorophore protoporphyrin IX exhibits strong fluorescence in gliomas compared to surrounding brain tissue under blue light. Intraoperative 5-ALA enables more complete resection of malignant glioma leading to greater survival (42–46).

#### Confocal Microscopy

Confocal microscopy combined with chemical-induced fluorescence may provide non-invasive histological images. This is also referred to as optical sectioning and allows visualization of the tumor in one plane. This technology reduces fluorescent light scatter, thus, resulting in higher resolution and contrast images, with *in vivo* magnification up to 1000× (47). Intraoperative visualization of brain tumors is greatly enhanced by new technologies of 5-ALA with confocal microscopy that is of importance not only for resection of high-grade gliomas but also for intraoperative visualization of anaplastic foci in a tumor initially suspected of being low grade (48). Fluoroscein injected intraoperatively and visualized with white light, use of fluorescein filters attached to the microscope, and confocal endomicroscopy demonstrate an increased success in near total removal of tumor. Intraoperative confocal microscopy is a safe, effective, and convenient method to visualize cellular 5-ALA-induced tumor fluorescence within LGGs and at the brain-tumor interface, which may aid in obtaining a greater extent of resection (49–51).

#### Microspectrofluorometer

Microspectrofluorometer measures the fluorescence spectra to distinguish tumor from normal tissue microscopically *in vivo*. As the 5-ALA method visualizes the tumor directly, the microspectrofluorometer utilizes a readout device (spectrometer or spectroscope) to differentiate the fluorescence emission from the glioma. This dye-free method identifies tumors based on the glioma’s unique autofluorescence that is distinct from normal brain. Under certain pathological processes, the autofluorescent properties of tissues change due to oncogenesis (52). Time-resolved fluorescence spectroscopy records the decay profiles of the autofluorescence that improves the sensitivity on tumor delineation. Under microspectrofluorometry, the normal white matter showed two peaks of fluorescence emission at 390 and 460 nm; the 390-nm emission peak was absent or reduced from the gliomas (53, 54). The spectrofluorometry method is accurate in differentiating between tumor and normal tissue. Tumor discrimination may be enhanced by certain agents, such as chloro-aluminum phthalocyanine tetrasulfonate (55).

#### Other Tumor Delineating Methods

**Figure 1:**
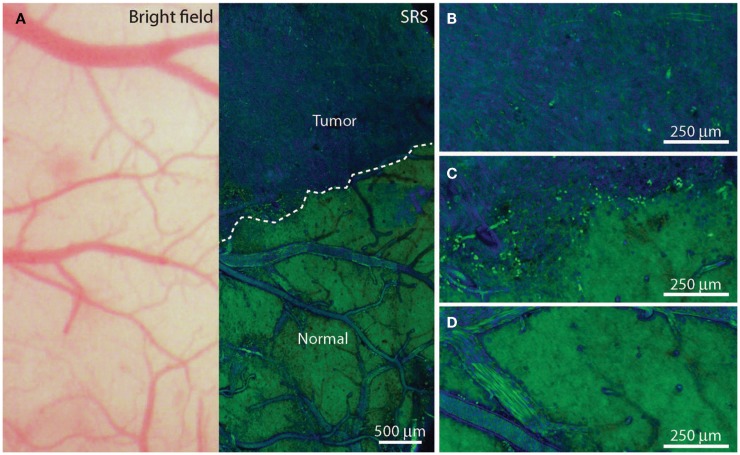
***In vivo* stimulated Raman scattering microscopy images of human glioblastoma multiforme xenografts**. **(A)** The arachnoidal and pial vessels on the surface of the normal brain were clearly identified in the standard bright field and stimulated Raman scattering images. Visualization of the tumor was undetectable under standard operative conditions. Certain regions of brain tissue that appeared grossly normal under bright field microscopy revealed extensive tumor infiltration on stimulated Raman scattering microscopy. The dashed line represents the tumor margin which was observed both biochemically and structurally. **(B,C)** The glioblastoma multiforme xenograft tissue was blue and cellular. **(D)** Normal axonal processes and vascular patterns were noted in non-infiltrated (normal) cortex. [Permission obtained from the publisher American Association for the Advancement of Science (AAAS); Ji et al. (63)].

Other tumor delineating methods: (1) *Fluorescence lifetime imaging microscopy (FLIM)* allows the demarcation of tumor from normal tissue. Tumor metabolism is different from that of normal tissue. At a wavelength band emission of 460 ± 25 nm corresponding to NADH/NADPH fluorescence, malignant gliomas exhibit a weaker fluorescence intensity (*p* < 0.05) and a longer duration (*p* < 0.005) (56). (2) *Tumor Paint BLZ-100* is a chlorotoxin ligand conjugated with indocyanine green that has a higher affinity for gliomas. The indocyanine green labeled tumor tissue can be identified vividly under near-infrared (NIR) lighting (57). (3) A *IRDye 800CW-RGD peptide* in tumor delineation serves as a probe to bind integrin receptors that are overexpressed in malignant gliomas for angiogenesis and growth. 800CW-RGD peptide-enhanced tumors may be visualized using near-infrared IRDye (58). (4) A *dye-loaded polyacrylamide nanoparticles coated F3 peptide* in tumor demarcation produces tight binding on the tumor surface receptor nucleolin. The affinity binding of dye-loaded nanoparticles with glioma helps to visualize the affected tissue from normal brain (59, 60). (5) *Triple-modality magnetic resonance imaging–photoacoustic Raman imaging nanoparticles* have been studied experimentally as a molecular strategy to delineate tumors. Intravenous injection of these nanoparticles into glioblastoma-bearing animals allows sharp tumor delineation due to specific retention of the nanoparticles by tumor cells (61). (6) *Stimulated Raman scattering (SRS)* microscopy can differentiate tumors from normal brain based on diverse Raman spectra, which reflects the histoarchitectural and biochemical variations of the tumor. SRS serves as a label-free technique for tumor demarcation *in vivo* by which the revealed tumor margins correlate well with histological assessment (kappa = 0.98) (Figure 1) (60, 62, 63). (7) Two-photon or multi-photon fluorescence microscopy has been utilized for tumor margin visualization. The advantage of using infrared lighting avoids the phototoxicity of tissues exposed to light in the surgical field (64).

## Discussion

The ultimate goal of brain-tumor surgery is to attain maximum tumor resection and to achieve the greatest survival with minimal neurological deficits. In addition, it is important to obtain a precise pathological diagnosis of the cellular origins and grade of the tumor. The surgery reduces the neurological symptoms caused by tumor compression/invasion and tumor-associated tissue edema. The key issue for glioma surgery is to obtain intraoperative tumor delineation. Since the texture and color of gliomas, especially low-grade tumors, are similar to normal brain tissue, accurate tumor identification remains a challenge for neurosurgeons. Traditionally, the determination of tumor margins depends on the experience of the neurosurgeon to sense subtle differences in color, texture, and surface vascularity of the glioma. Several technologies used intraoperatively to delineate tumor margins have been developed and continue to rapidly evolve. Current available technologies that assist with intraoperative demarcation between tumor and normal brain tissue are listed in Table 1.

**Table 1:** **Intraoperative demarcation between tumor and normal brain tissue**.

Methods	Suitability	Characteristics
Image-based navigation	Plan the surgical approach to biopsy the lesion; attempt gross tumor resection	Provides information of tumor size, shape, and location; not a true real-time tool^a^
Intraoperative tissue sampling	Confirm tumor diagnosis; possibly identify tumor origin and grade	High-diagnostic value; specimens are piecemeal and discontinuous; must combine multiple specimens; often not real-time
Electrophysiological monitoring	Identify important functional regions and circuits; minimize neurologic deficits	Indirect tumor delineation via the responses or feedback of awake patients; real-time
Enhanced visual tumor demarcation^b^	Identify tumor remnants or infiltrating tumors to obtain complete resection	Strong correlation with the surgeon’s view of the field; real-time

*^a^MRI information for navigation is not real-time*.

*^b^Microspectrofluorometer is similar to the method of enhanced visual tumor demarcation by using a readout device to detect the fluorescence difference instead of visualization*.

Each innovative technology has its advantages and limitations. In a recent review, advanced intraoperative delineation techniques have greatly improved the extent of glioma resection compared to conventional methods (42). Intraoperative imaging with CT, US, and MRI navigation identifies tumor location and extension of the gliomas and are used to assist in planning the surgical approach. These methods do not demonstrate residual tumor or tumor infiltration. Enhanced visual tumor demarcation techniques do not provide images of the entire tumor but do demonstrate residual tumor in the surgical field. This technology requires high signal-to-noise ratio or tumor-to-brain fluorescence ratio in the surgical resection cavity (65). Using the 5-ALA method, the tumor-to-brain fluorescence ratio is acceptable (66, 67). In the future, improved methods with a greater tumor-to-brain fluorescence ratio will be developed such as CLR1502 (68).

Ideal methods to differentiate gliomas from normal brain include (1) easy to perform and accurate, (2) real-time that is concordant with a surgeon’s view and may be monitored continuously, (3) maximal functional protection, (4) minimal technical challenges, and (5) cost-effective. Neuronavigation is based on imaging information to develop and optimize the surgical approach to the tumor while providing an aggressive and safe tumor debulking using methods of demarcation, such as 5-ALA, to identify residual tumor. Neurophysiologic functional monitoring may prove beneficial in avoiding damage to eloquent areas. Mastering and properly applying methods to discriminate gliomas from normal brain tissue in standard neurosurgical practice will increase the success of total glioma excision.

## Author Contributions

ZZ, LS, DS, YZ, MH, and CS substantially contributed to the writing, editing, and reviewing of the manuscript of this mini-review. All of the authors have approved the final version of the manuscript.

## Conflict of Interest Statement

The authors declare that the research was conducted in the absence of any commercial or financial relationships that could be construed as a potential conflict of interest.
